# An unusual cause of new-onset ascites and apparent severe acute kidney injury

**DOI:** 10.1590/2175-8239-JBN-2022-0011en

**Published:** 2022-06-27

**Authors:** João Oliveira, Miguel Costa, Joana Freitas, Inês Sala, Sofia Santos, António Cabrita

**Affiliations:** 1 Universitário do Porto, Centro Hospitalar, Departamento de Nefrologia, Porto, Portugal; 2 Unidade Local Saúde Alto Minho, Departamento de Medicina Interna, Viana do Castelo, Portugal

**Dear Editor,**

We present the case of a 77-year-old man admitted to the emergency department with acute abdominal pain. He had a medical history of metabolic syndrome, diabetes mellitus, generalized atherosclerotic disease, stage 2 chronic kidney disease (eGFR CKD-EPI 61 mL/min/1.73 m^2^, Cr 1.4 mg/dL), and a past medical history of high-grade non-invasive papillary urothelial cancer submitted to transurethral resection two years ago and local chemotherapy with mitomycin C. He was on surveillance with regular cystoscopies without evidence of relapse. He had a bladder with normal capacity and focal areas of fibrosis.

**Figure 1:**
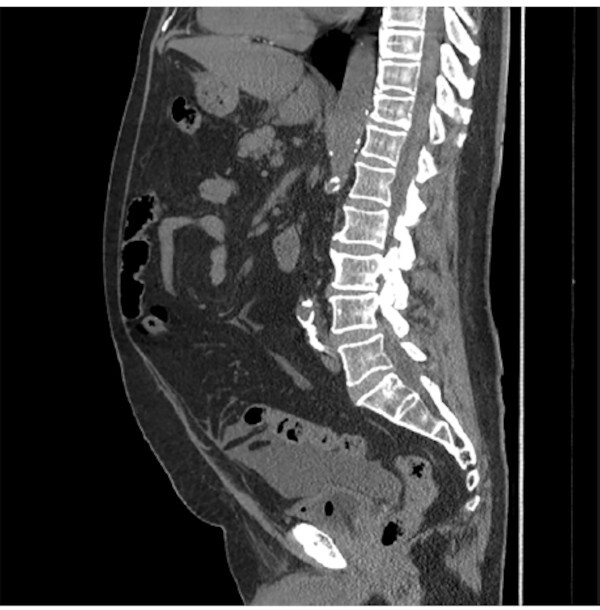
Non-contrast CT-scan with intraperitoneal fluid compatible with ascites.

Four days earlier, he suffered an acute pain in the low abdomen without a precipitating event. Since then, he had been feeling unwell and complaining of decreased urinary output. On admission, he had discomfort in the low abdomen with no other signs or symptoms including evidence of hepatic or cardiac disease. His physical examination was normal aside from mild increase in abdominal girth with diffuse discomfort but without evidence of peritoneal irritation. Lab work was positive for elevated serum creatinine of 7.8 mg/dL and serum urea of 165 mg/dL. Surprisingly, he had a serum cystatin C of 1.41 mg/dL, potassium of 5.2 mEq/L, and bicarbonate HCO_3_ of 22 mM. His hepatic profile and pro-BNP level were normal. Abdominal ultrasound and non-contrast CT scan (Figure 1) showed moderate amount of free ascites and no evidence of hepatic abnormalities. Renal ultrasound excluded hydronephrosis. His bladder was moderately full. Paracentesis was performed and a transparent pale yellow fluid with elevated creatinine (ascites to serum ratio of 2:1) and potassium values (ascites to serum ratio of 4:1) were diagnostic of urinoma (Table 1 - analytical workup and evolution).

Urinomas are urinary collections, usually related to trauma or surgical complications. Patients with bladder fragility are also at increased risk. The pathophysiology behind the analytical alterations in this case relates to the peritoneal capacity for absorption of creatinine and urea and for metabolization of cystatin C by the kidney tubules (which explains why increased serum creatinine and normal cystatin C levels were observed). Cystatin C is a low molecular weight protein produced by all nucleated cells and filtered but not reabsorbed by the glomerulus. However, it is metabolized in the tubules. It is usually used as an alternative marker of kidney function as it is believed to have a relatively constant rate of production and is not affected by changes in diet, gender, age, or muscle mass^1-5^. If the patient had presented to the emergency department on the first day of symptoms, the ascites to serum creatinine and potassium ratios would have been much higher (diluting effect). If the patient really had severe acute kidney injury with oliguria for four days, alterations in acid-base and serum electrolytes would be expected. In this case, the kidneys were functioning properly, but urine was being diverted into the abdomen. The risk associated with urinoma formation is the development of chemical peritonitis. The definite diagnosis is established by bladder CT scan (with urethral contrast), cystoscopy or intraoperative evaluation. Initial priorities include removal of renal/bladder obstruction (if present), antibiotic prophylaxis, and stopping urine leakage. The approach to repairing a bladder injury depends on the site, type, and extent of the injury^6^. Although rare, similar cases have been reported^1-4^.

The patient presented a gradual improvement of kidney function with conservative management and was discharged with a bladder catheter (Table 1).

**Table 1:** Analytical workup and evolution at baseline, admission, 1 week evolution, and post-nephrectomy, ureter and partial bladder excision

		3 months prior	Admission	At 24h	At 48h	1 week later	After surgery
**Hematology**							
WBC	4.00 - 11.00 x10^3/µL		10.3				
Hemoglobin	13 - 17 g/dL		12.6				
Platelets	150 - 400 x10^3/µL		400				
**Blood Chemistry**							
Creatinine	0.7 - 1.2 mg/dL	1.4	7.8	**4.55**	2.03	1.36	2
Urea	10 - 50 mg/dL	46	165	134	78	49	43
Cystatin C	0.62 – 1.11 mg/L	1.33	1.41				
Glomerular filtration (Larsson)	> 90 mL/min	54	50				
Total bilirubin	0.20 - 1.00 mg/dL		0.24				
Aspartate aminotransferase	10 - 34 U/L a 37°		19				
Alanine aminotransferase	10 - 44 U/L a 37°		18				
Alkaline phosphatase	40 - 129 U/L a 37°		51				
Gama-glutamil transferase	10 - 66 U/L a 37°		63				
Lactate dehydrogenase	135 - 225 U/L a 37°		132				
Pro-BNP	0 - 125 pg/mL		46.5				
Albumin	3.4 - 4.8 g/dL		4.32				
Bicarbonate	22 - 26 mmol/L		21.8			21.4	
Sodium	135 - 145 mmol/L		136	137	140	133	138
Potassium	3.50 - 5.00 mmol/L		5.27	5.01	4.73	4.3	4.23
Chloride	95 - 105 mmol/L		104	105	106	103	101
Reactive C protein	0.0 - 5.0 mg/L		14.72				
**Urine**							
Color	Yellow		Yellow				
Appearance	Clear		Clear				
pH	4.8 - 7.4		5				
Relative density	1.015 - 1.025		1.019				
Leukocyte esterase	Leu/µL		25				
Protein	mg/dL	30	15				
Nitrites	Negative		Negative				
Glucose	0 - 15 mg/dL		0				
Bilirubin	0.0 - 0.2 mg/dL		0				
Hemoglobin	0.00 - 0.01 mg/dL		0.06				
**Urine sediment**							
WBC	0 - 2/HPF		10 to 25				
RBC	0 - 2/HPF		2 to 5				
Squamous cells	0 - 5/Field 100 X		5 to 10				
Casts	0 - 0/Field 100 X		0 to 2				
Protein-to-creatinine ratio	0.015 - 0.068 g/g crea	0.171	0.015				
Urine microbiology	Sterile		Sterile				
**Peritoneal fluid**							
pH	6.8 - 7.6			8			
Density	1.010 - 1.030			1.013			
Lactate dehydrogenase	U/L			53			
Glucose	mg/dL			94			
Protein	0.30 - 4.10 g/dL			1.57			
Albumin	g/dL			1.16			
Creatinine	mg/dL			**9.49**			
Potassium	mmol/L			**20.07**			
**Cytology**							
RBC	0 - 100000 cell/µL			200			
WBC	0 - 500 cell/µL			290 (mononuclear predominance)			
Other cells	Cell/µL			85			
Peritoneal microbiology	Sterile			Sterile			

At consultation, urinary cytology was unremarkable, and cystoscopy did not detect a bladder leak. A CT-scan with systemic contrast identified a left kidney nodule with contrast uptake suggestive of advanced urothelial cancer. He was submitted to laparoscopic nephrectomy, urethrectomy, and partial bladder excision. At that moment, a bladder leak was identified and corrected. The patient had a bladder with fibrotic changes (scarring) and focal loss of elasticity, secondary to bladder cancer and respective treatment. We believe this was the cause of the urinoma.
